# Case Report: successful non-surgical management of massive isolated tricuspid Libman-Sacks endocarditis in a pregnant patient with SLE and secondary APS

**DOI:** 10.3389/fimmu.2026.1858060

**Published:** 2026-05-28

**Authors:** Jiaoniu Duan, Lin Han, Wenli Zhao, Gailian Zhang

**Affiliations:** 1 The Rheumatology and Immunology Department of Shanxi Provincial People’s Hospital, Shanxi Medical University, Taiyuan, China; 2 The Ultrasound Department of Shanxi Provincial People’s Hospital, Shanxi Medical University, Taiyuan, China

**Keywords:** antiphospholipid syndrome, Libman-Sacks endocarditis, pregnancy, systemic lupus erythematosus, thrombocytopenia, tricuspid valve

## Abstract

**Background:**

Isolated tricuspid valve Libman-Sacks endocarditis (LSE) is extremely rare, and its occurrence during pregnancy with severe thrombocytopenia poses major diagnostic and therapeutic challenges.

**Case summary:**

A 21-year-old pregnant woman at 13 weeks of gestation with systemic lupus erythematosus (SLE) and secondary antiphospholipid syndrome (APS) presented with severe thrombocytopenia (platelet count 21 × 10^9^/L). Echocardiography revealed two isolated tricuspid vegetations (the largest measuring 24.1 × 19.3 mm). She declined surgery and insisted on continuing the pregnancy with medical management. Treatment included pulse methylprednisolone, plasma exchange, intravenous immunoglobulin, cyclosporine, hydroxychloroquine, and anticoagulation. Anticoagulation was initiated after partial platelet recovery. The patient responded favorably, with platelet normalization, progressive vegetation regression, and successful delivery of a healthy infant. Echocardiography at nine months showed near-complete regression of the larger vegetation and complete resolution of the smaller one, with normal valve function.

**Conclusion:**

This case demonstrates that intensive immunomodulatory therapy combined with timely anticoagulation after partial platelet recovery can achieve vegetation regression and avoid cardiac surgery in pregnant patients with SLE/APS and massive isolated tricuspid LSE. To our knowledge, this is the first reported case of non-surgical management of this life-threatening condition.

## Introduction

Systemic lupus erythematosus (SLE) is a chronic autoimmune disease characterized by the production of antinuclear antibodies and multisystem involvement, predominantly affecting women of childbearing age (1). Antiphospholipid syndrome (APS), defined by persistent antiphospholipid antibodies (aPLs), may occur as a primary condition or secondary to SLE and manifests as thromboembolic events and/or pregnancy morbidity (2). Notably, aPLs positivity is detected in 30–40% of individuals with SLE (3). The coexistence of SLE and APS is associated with more severe disease manifestations and significantly increased risks of adverse pregnancy outcomes, including miscarriage, preeclampsia, fetal growth restriction, and preterm birth (4).

Cardiac involvement is common in SLE, affecting up to 50% of patients, with manifestations ranging from pericarditis and myocarditis to valvular heart disease (5). Among these, Libman-Sacks endocarditis (LSE) is an established non-bacterial thrombotic endocarditis characterized by sterile vegetations on cardiac valves (6). The mitral and aortic valves are most commonly affected, whereas isolated tricuspid valve involvement is exceptionally rare (5, 7). The pathogenesis of LSE is driven by immune-mediated endothelial injury and thrombosis (5, 8). SLE-related chronic inflammation and autoantibody deposition play a central role in initiating valvular damage, which is further amplified by the hypercoagulable state of APS when present (8).

The 2023 ACR/EULAR APS classification criteria incorporate both cardiac valve involvement and thrombocytopenia as clinical domains to capture and quantify the heterogeneous manifestations of APS (2). Notably, thrombocytopenia in APS patients has been closely linked to LSE (9). A retrospective study comparing 11 SLE patients with LSE to 29 SLE controls found that the prevalence of antiphospholipid antibodies, particularly triple positivity, was markedly higher in LSE patients (72.7% vs. 13.8%) (10). Given that secondary APS is a well-established predictor of adverse pregnancy outcomes in women with SLE (11), the management of pregnant SLE/APS patients with LSE and thrombocytopenia presents unique challenges. Severe thrombocytopenia further complicates management: anticoagulation is necessary to prevent embolism from large vegetations but significantly increases bleeding risk. Notably, although thrombocytopenia is often perceived as a bleeding risk, evidence suggests that APS patients with thrombocytopenia may have a paradoxical increase in thrombotic risk. However, current guidelines lack specific guidance for managing isolated tricuspid LSE during pregnancy in patients with SLE/APS, particularly regarding the optimal timing of anticoagulation initiation in the setting of severe thrombocytopenia.

Herein, we report a case of a pregnant woman with SLE and secondary APS who presented with severe thrombocytopenia and a large isolated tricuspid valve vegetation with a second smaller lesion. Through intensive immunomodulatory therapy and a carefully balanced anticoagulation strategy, we achieved significant vegetation regression and favorable maternal and fetal outcomes without cardiac surgery, highlighting a potential non-surgical approach for this rare and challenging condition.

## Case presentation

### History

A 21-year-old woman was admitted on July 7, 2025, with a chief complaint of alopecia and photosensitivity for two years, joint swelling and pain for five days, and generalized petechiae and ecchymosis for three days. Approximately two years ago, she developed non-scarring diffuse alopecia predominantly involving the vertex, accompanied by photosensitivity with facial erythema upon sun exposure, without xerostomia, xerophthalmia, arthralgia, foamy urine, Raynaud’s phenomenon, or oral/genital ulcers. Five days before admission, she developed symmetric swelling and pain of both wrists with morning stiffness lasting more than 30 minutes, without involvement of other joints, and denied fever, chills, or night sweats. Three days before admission, she developed widespread petechiae and ecchymosis, initially affecting the lower limbs and subsequently spreading to the trunk and upper extremities, without gingival bleeding, epistaxis, or melena. On the same day, routine antenatal blood tests at our hospital’s obstetric clinic revealed severe thrombocytopenia (platelet count 21 × 10^9^/L), prompting hospitalization. Since the onset of symptoms, her appetite, sleep, and bowel habits had remained normal, with no change in body weight. Her past medical, personal, and family histories were unremarkable. She married at age 20 (gravidity 2, parity 0). At 20 weeks of gestation in 2024, she underwent termination of pregnancy due to fetal demise, with concurrent laboratory testing showing a platelet count of 99 × 10^9^/L that was not further investigated. Upon admission, she was in her second pregnancy, naturally conceived, at 13 weeks of gestation. Her spouse was reported to be healthy.

### Physical examination

Upon admission, vital signs were stable: temperature 36.5 °C, pulse 98 beats/min, respiratory rate 20 breaths/min, blood pressure 112/77 mmHg. Widespread petechiae and ecchymosis were observed over the trunk and extremities, predominantly on the lower limbs, with variable sizes, partially confluent, and non-blanching. Hair was sparse with non-scarring diffuse alopecia, most pronounced on the vertex. No malar rash or oral ulcers were noted. Lung auscultation revealed clear breath sounds without crackles or wheezes. Cardiac auscultation revealed a regular rhythm at 98 beats/min, with a grade 2/6 holosystolic murmur best heard at the left sternal border (4th intercostal space). The murmur increased in intensity during inspiration (Carvallo’s sign) and decreased during expiration. No pericardial friction rub was audible. The abdomen was slightly distended, with the liver and spleen not palpable below the costal margin. Musculoskeletal examination revealed swelling and tenderness of both wrists with mild limitation of flexion and extension. No lower extremity edema was noted.

### Laboratory findings

Laboratory tests on admission revealed a white blood cell count of 6.00 × 10^9^/L, hemoglobin 120 g/L, and severe thrombocytopenia with a platelet count of 22 × 10^9^/L (reference range 125-350). Urinalysis and stool examination were unremarkable. Liver and kidney function tests were within normal limits. Coagulation studies showed a prolonged activated partial thromboplastin time of 59.9 seconds (reference range 25.1-36.5), with normal prothrombin time, international normalized ratio, and fibrinogen; D-dimer was elevated to 353 ng/ml. Inflammatory markers were elevated: erythrocyte sedimentation rate (ESR) 48 mm/h and C-reactive protein (CRP) 9.69 mg/L. Immunological studies revealed low complement C3 (0.83 g/L, reference range 0.90-1.80) with normal C4, and elevated immunoglobulin IgG (29.69 g/L) with normal IgA and IgM. Autoantibody testing was positive for antinuclear antibodies (1:1000, speckled pattern), weakly positive for anti-Ro52 and anti-AMA-M2 antibodies, and positive for anti-double-stranded DNA antibodies. aPLs were measured by chemiluminescence immunoassay (CLIA) and were markedly elevated at baseline: anticardiolipin (aCL) IgA 24.50 CU, aCL IgG 820.50 CU, anti-β2 glycoprotein I (aβ_2_GPI) IgA 21.30 CU, and aβ_2_GPI IgG 5456.20 CU (cutoff value for all assays: < 20.00 CU, with values ≥ 20.00 CU considered positive). Repeated testing using the same CLIA method after 12 weeks confirmed persistent positivity of aCL IgG and aβ_2_GPI IgG antibodies (**Table 1**), although titers had declined substantially following intensive immunomodulatory therapy (aCL IgG from 820.50 to 51.8 CU; aβ_2_GPI IgG from 5456.20 to 218.7 CU). Lupus anticoagulant (LA) testing was not performed due to local unavailability. All other antibodies tested (including ANCA, anti-GBM, rheumatoid factors, and anti-CCP) were negative. Blood cultures (three sets, drawn from separate venipuncture sites) were negative for bacterial and fungal growth. Serologic tests for infectious diseases, including HIV, syphilis (Treponema pallidum), hepatitis B surface antigen, and hepatitis C antibody, were all negative. Antistreptolysin O (ASO) titer was within the normal range. Serial changes in platelet count, serological markers, and vegetation size are summarized in **Table 1**.

**Table 1:** Dynamic evolution of laboratory markers, vegetation size, and treatment modifications over time.

Timepoint	PLT (×10^9^/L)	ESR (mm/h)	CRP (mg/L)	IgG (g/L)	C3 (g/L)	C4 (g/L)	aCL IgG (CU)	aβ_2_GPI IgG (CU)	Vegetation size (mm)	Treatment modifications clinical status
Day1	22	48	9.69	29.69 ↑	0.83	0.16	820.5	5456.2	24.1×19.3, 7.5x8.1	MP 80 mg IV QD ×3d, IVIG 20 g QD ×5d
Day3	36	–	–	–	–	–	–	–	–	MP 80 mg IV QD ×3d, IVIG 20 g QD ×5d, HCQ 0.2 g BID, Dalteparin 5k IU Q12H
Day5	43	–	–	–	–	–	–	–	–	MP 500 mg IV QD ×3d, IVIG 20 g QD ×5d, HCQ 0.2 g BID, CsA 75 mg BID, Dalteparin 5k IU Q12H
Day8	63	27	<5	–	–	–	–	–	18.9x11.5, 5. 3x4.2	MP 80 mg IV QD, HCQ 0.2 g BID, CsA 75 mg BID, Dalteparin 5k IU Q12H, LDA 100 mg QD, PLEX ×5d
Day11	107	–	–	13.17	1.03	0.22	71.9	343.5	–	Continue same
Week 2	164	15	<5	–	–	–	–	–	12.8x12.8, 6.4×3.5	Pred 60 mg QD, HCQ 0.2 BID, CsA 75 mg BID, Dalteparin 5k IU Q12H, LDA 100 mg QD
Month 1	160	9	<5	10.94	1.4	0.18	94.9	418.8	12.0×12.3, 5.0x4.1	Pred 55 mg QD, HCQ 0.2 BID, CsA 75 mg BID, Dalteparin 5k IU Q12H, LDA 100 mg QD
Month 3	203	11	<5	12.56	1.64	0.21	51.8	218.7	11.2×7.1, 7.9x4.8	Pred 35 mg QD, HCQ 0.2 BID, CsA 75 mg BID, Dalteparin 5k IU Q12H, LDA 100 mg QD
Month 6	199	8	<5	13.32	1.09	0.17	40.9	94.6	9.3×4.5, 5.1×2.9	Pred 15 mg QD, HCQ 0.2 BID, CsA 75 mg BID, Dalteparin 5k IU Q12H, LDA 100 mg QD
Month 9	236	6	<5	15.87	1.21	0.23	32.4	71.3	7.9×3.0 (smaller resolved)	Pred 10 mg QD, HCQ 0.2 BID, CsA 75 mg BID, LDA 100 mg QD

PLT, platelet count; ESR, erythrocyte sedimentation rate; CRP, C-reactive protein; aCL, anticardiolipin; aβ_2_GPI, anti-β2-glycoprotein I; C3, complement C3; C4, complement C4; MP, methylprednisolone; HCQ, hydroxychloroquine; Pred, Prednisone; CsA, cyclosporine A; LDA, low-dose aspirin; PLEX, plasma exchange; IVIG, intravenous immunoglobulin.

### Imaging studies

Obstetric ultrasound demonstrated an intrauterine singleton pregnancy at 13 weeks of gestation. Transthoracic echocardiography (TTE) showed two irregular, echogenic, mobile masses attached to the tricuspid valve: one measuring 24.1 × 19.3 mm on the atrial side of the posterior leaflet and another measuring 7.5 × 8.1 mm on the ventricular side of the anterior leaflet. Forward flow across the tricuspid valve was normal, but moderate regurgitation was present, with an estimated pulmonary artery systolic pressure of 46 mmHg, indicating mild pulmonary hypertension. The remaining valves and the aorta and pulmonary artery were unremarkable. Abdominal ultrasound and lower extremity venous ultrasound showed no abnormalities. Serial echocardiographic findings are summarized in **Table 1**.

### Diagnosis

Based on the 2019 EULAR/ACR classification criteria (total score 23), the diagnosis of SLE was confirmed. APS was diagnosed based on persistent positivity of aCL IgG and aβ_2_GPI IgG antibodies on repeated testing 12 weeks apart, combined with thrombocytopenia and a history of unexplained fetal demise, satisfying both the laboratory and clinical domains of the 2023 ACR/EULAR APS classification criteria (2). The differential diagnosis of the tricuspid valve vegetations included infective endocarditis, cardiac myxoma, and rheumatic heart disease. Infective endocarditis was excluded by the absence of fever, three sets of negative blood cultures drawn before any antibiotic exposure, and the lack of valvular destruction (no perforation or disruption). Formal assessment using the modified Duke criteria yielded only one major criterion (echocardiographic vegetation) and no minor criteria, classifying the case as ‘rejected’ for infective endocarditis. Although transesophageal echocardiography (TEE) is generally considered superior to TTE for detecting small valvular vegetations, TTE in this case provided high-quality images that clearly delineated both tricuspid vegetations and their mobility. Given these clear TTE findings, the low clinical suspicion for infective endocarditis, and the procedural risks of TEE during pregnancy, transesophageal echocardiography was deemed not clinically imperative and was not pursued. Serial TTE was used throughout the treatment course to monitor vegetation regression. Cardiac myxoma was considered unlikely because myxomas are typically solitary, pedunculated, and located in the left atrium; in contrast, the vegetations in this patient were multiple, irregular, and attached to the tricuspid valve leaflets. Rheumatic heart disease was ruled out by the absence of a history of rheumatic fever, a normal ASO titer, and the lack of valvular fusion or predominant stenosis. Given the patient’s underlying SLE/APS, the absence of a history of rheumatic fever or infective endocarditis, and the presence of high-titer aPLs, a diagnosis of LSE (isolated tricuspid valve involvement) was established. The patient’s SLE disease activity index (SLEDAI-2K) score was 11, indicating moderate disease activity by standard criteria; however, the presence of a large tricuspid vegetation (a severe cardiac manifestation not reflected in the score) and severe thrombocytopenia (lowest platelet count 21 × 10^9^/L) pointed to a life-threatening condition.

### Multidisciplinary assessment and treatment decision

A multidisciplinary team (MDT) comprising rheumatologists, obstetricians, cardiac surgeons, hematologists, and ultrasonographers was assembled to evaluate maternal and fetal risks. The cardiac surgery team highlighted that detachment of the tricuspid vegetation could cause life-threatening pulmonary embolism, recommending low-molecular-weight heparin with close monitoring, as surgery during pregnancy was extremely hazardous. The obstetrics team noted the risks of intracranial or visceral hemorrhage, emphasized that severely active disease significantly increased the likelihood of adverse pregnancy outcomes, and advised termination of pregnancy after the platelet count reached 70 × 10^9^/L, with aggressive control of the underlying condition if the pregnancy continued. The ultrasonography team noted that although isolated tricuspid LSE is extremely rare, the morphological features of the vegetations—irregular, loosely structured, attached to the valve leaflets without destruction—combined with the patient’s history of SLE/APS, strongly supported the diagnosis; they also recommended serial echocardiography to monitor vegetation changes during follow-up. The rheumatology and hematology teams emphasized prompt intensive immunosuppression for active severe SLE with severe thrombocytopenia, with active monitoring of clinical improvement in bleeding symptoms, platelet counts closely monitored to guide the timing of anticoagulation, which should be initiated as early as safely possible. After thorough counseling on the life-threatening risks, the patient and her family remained committed to continuing the pregnancy. Consequently, a non-surgical strategy was finalized, combining intensive immunosuppression with anticoagulation.

### Treatment and outcomes

**Figure 1:**
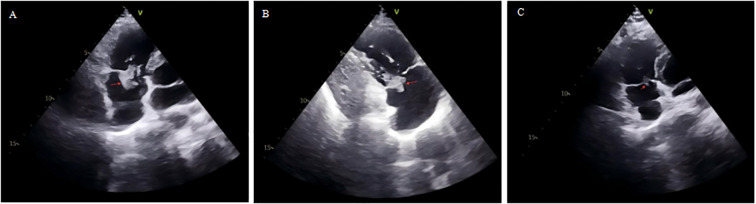
Serial echocardiographic images showing regression of the tricuspid vegetation. **(A)** At baseline: a large vegetation measuring 24.1 × 19.3 mm. **(B)** After one month of treatment: the vegetation decreased to 12.0 × 12.3 mm. **(C)** After nine months of treatment: the vegetation regressed to 7.9 × 3.0 mm with preserved valve function.

Induction therapy included glucocorticoids, intravenous immunoglobulin (IVIG, 20 g/day for five days), cyclosporine (CsA, 75 mg twice daily), hydroxychloroquine (HCQ, 0.2 g twice daily), and five sessions of plasma exchange (PLEX). Glucocorticoids were given as initial methylprednisolone (MP) 80 mg/day for three days, followed by pulse MP 500 mg/day for three days, then tapered. After three days of MP 80 mg/day (concurrent with IVIG), the platelet count rose from 22 × 10^9^/L to 36 × 10^9^/L, and no new petechiae or ecchymoses were observed, indicating cessation of active bleeding. Given the imminent risk of life-threatening pulmonary embolism from the large, mobile vegetation (24.1 × 19.3 mm), the MDT judged that the thromboembolic risk outweighed the bleeding risk, and dalteparin was cautiously initiated at a therapeutic dose of 5000 IU subcutaneously twice daily before the platelet count reached the conventional safety threshold of 50 × 10^9^/L. Subsequently, during the three-day pulse MP therapy, IVIG was continued for the first two days. On Day 8, the platelet count rose to 63 × 10^9^/L, at which point low-dose aspirin (LDA, 100 mg/day) was added and five sessions of PLEX were initiated. The PLEX subsequently normalized the platelet count. The patient responded favorably, with normalization of platelet count, anti-dsDNA antibodies becoming negative, erythrocyte sedimentation rate and C-reactive protein returning to normal levels, complement C3 increasing to the normal range, marked reduction in aCL and anti-β_2_GPI antibody titers, along with progressive regression of the tricuspid vegetation (**Table 1**). She continued the pregnancy without bleeding or thromboembolic complications. Fetal well-being was monitored with serial obstetric ultrasound every four weeks to assess growth, amniotic fluid volume, and Doppler velocimetry. From 28 weeks of gestation, antenatal testing including non-stress tests was performed twice weekly. All fetal assessments remained within normal limits throughout the pregnancy. Maintenance therapy consisted of tapering prednisone, CsA (75 mg twice daily), HCQ (0.2 g twice daily), dalteparin (5000 IU subcutaneously twice daily), and LDA (100 mg/day). At 31 weeks, the patient presented with a sudden gush of clear vaginal fluid, and a clinical diagnosis of preterm premature rupture of membranes (PPROM) was confirmed. Antenatal corticosteroids were administered for fetal lung maturation, and magnesium sulfate was given for fetal neuroprotection and tocolysis. Subsequent ultrasound revealed oligohydramnios, raising concern for potential fetal compromise. Given the increased risk of intra-amniotic infection, the concern for fetal compromise, and the need to facilitate precise control of peripartum anticoagulation, the decision was made to proceed with emergency cesarean section. To minimize hemorrhagic risk, dalteparin was discontinued 24 hours before the procedure. She delivered a healthy female infant weighing 1600 g, with Apgar scores of 8 and 9 at 1 and 5 minutes, respectively. Postoperatively, uterine tone, vaginal bleeding, and vital signs were closely monitored. At 12 hours after surgery, with hemodynamic stability, satisfactory uterine contraction, and no evidence of active bleeding, both dalteparin and LDA were resumed and continued throughout the postpartum period. At the final follow-up in March 2026 (four months postpartum, nine months after treatment initiation), echocardiography demonstrated near-complete regression of the larger vegetation (7.9 × 3.0 mm) and complete resolution of the smaller one, with normal valve function, at which time dalteparin was discontinued. The patient remained stable on tapering prednisone (10 mg/day) with CsA, HCQ, and LDA. Representative TTE images at baseline, after one month, and after nine months of treatment are presented in **Figure 1**.

## Discussion

LSE is a well-recognized non-bacterial thrombotic valvular complication that affects approximately 10% of patients with SLE, with the mitral and aortic valves being the predominantly involved sites (5). Isolated tricuspid valve involvement is an extremely rare presentation: a cross-sectional echocardiographic study of 342 SLE patients conducted by Moyssakis et al. detected 38 cases of LSE, among which only one case had isolated tricuspid involvement (12). Similarly, a retrospective study from China including 23 LSE patients reported only one case of tricuspid valve involvement (13). We report a 21-year-old pregnant woman at 13 weeks of gestation with SLE and secondary APS who presented with severe thrombocytopenia (platelet count 21 × 10^9^/L) and two isolated tricuspid vegetations (the largest measuring 24.1 × 19.3 mm). Through intensive immunomodulatory therapy (including PLEX, glucocorticoid pulse, IVIG, and pregnancy-safe immunosuppression) combined with timely balanced anticoagulation, we achieved significant vegetation regression and favorable maternal-fetal outcomes without cardiac surgery. To the best of our knowledge, this is the first reported case of non-surgical management of isolated tricuspid LSE with massive vegetations in a pregnant patient with SLE and secondary APS.

The precise mechanisms underlying LSE in SLE remain incompletely understood. However, current evidence suggests that the pathogenesis is driven by immune-mediated endothelial injury followed by subendothelial deposition of circulating immune complexes, which subsequently triggers the formation of sterile platelet-fibrin thrombi (14). In patients with concomitant SLE and APS, aPLs may promote a prothrombotic state via complement activation and platelet aggregation (3, 4). A meta-analysis by Zuily et al. demonstrated that among SLE patients, those with aPLs have a significantly higher risk of heart valve disease compared with those without aPLs, with valve thickening and vegetations being the most frequently reported abnormalities (15). This “double-hit” pathological process was clearly evident in our patient, a pregnant woman with concurrent SLE and secondary APS, who presented with severe thrombocytopenia, moderate disease activity by SLEDAI-2K with life-threatening hematologic and cardiac manifestations, and two isolated tricuspid vegetations (the largest measuring 24.1 × 19.3 mm). The hypercoagulable state inherent to pregnancy likely further amplified thrombotic risk, contributing to the exceptionally large vegetation size.

The 2025 ACR guidelines for SLE conditionally recommend anticoagulation and/or immunosuppressive therapy for LSE, though without specific guidance on regimen selection, treatment duration, or monitoring (1). Clinicians and surgeons generally advise surgical treatment for LSE complicated by significant valvular dysfunction, large vegetations, or embolization. However, valve surgery in SLE patients carries substantially higher risks, with a review of two surgical series reporting 3–5 times higher morbidity (25-30%) and mortality (13-25%) in SLE patients versus non-SLE populations (16, 17). Given these high risks, non-surgical alternatives warrant consideration. Indeed, a longitudinal study of 17 SLE patients with LSE and acute cerebrovascular disease found that after 6 months of anti-inflammatory and anticoagulation, valve vegetations decreased in size, regurgitation improved, and the majority of patients showed overall clinical improvement (18). In our patient, who was pregnant with moderate SLE disease activity but life-threatening thrombocytopenia and valvular involvement, surgical risk was prohibitively high. Therefore, we developed a conservative strategy combining intensive immunomodulation and anticoagulation through multidisciplinary collaboration, with close monitoring by serial laboratory tests and echocardiography.

A comprehensive review of the literature over the past three decades identified 17 reported cases of isolated tricuspid LSE (19–35) (**Table 2**). Among the 17 reported cases, the majority were female (16/17, 94.1%). SLE was present in 11 cases (64.7%), including 6 with secondary APS. Primary APS was present in 6 cases (35.3%). Most patients (11/17, 64.7%) underwent surgical valve replacement or repair, reflecting a prevailing tendency toward surgical intervention. Only six cases (35.3%) were managed with medical therapy alone, including anticoagulation and immunosuppression (21–23, 27, 32, 34). Of these six, all vegetations were substantially smaller than the mass in our patient; none had severe thrombocytopenia or active bleeding, and none were pregnant. Only one previously reported case of tricuspid LSE occurred during pregnancy. Migliorini et al. described a 38-year-old primigravida with primary APS who developed a 2 × 1.5 cm tricuspid vegetation at 15 weeks of gestation. After two weeks of unsuccessful anticoagulation, she underwent surgical vegetation resection and bioprosthetic valve replacement at 16 weeks, ultimately delivering a live infant by cesarean section at 35 weeks (33).

**Table 2:** Reported cases of isolated tricuspid Libman-Sacks endocarditis (1996–2026).

Author	Year	Age/Sex	Underlying disease	Clinical presentation	Primary treatment	Outcome
Chan-Lam et al. (19)	2001	29/F	APS	Dyspnea, chest pain, RV outflow tract mass	Mass excision	Not specified
Falode et al. (20)	2006	35/F	APS	Massive right atrial vegetation	Valve replacement	Not specified
Zurick et al. (21)	2007	38/F	SLE, APS	Paradoxical embolism	Medical (anticoagulation)	Stable
Moaref et al. (22)	2010	29/F	SLE	Cardiac murmur	Medical (Pred + HCQ)	Stable at 3 years
Raj et al. (23)	2013	10/F	SLE	Chorea	Medical (steroids + anticoagulation)	Chorea improved
Gur et al. (24)	2014	20/F	SLE	Tricuspid stenosis	Commissurotomy + annuloplasty	Asymptomatic at 18 months
Wang et al. (25)	2014	40/F	SLE	Tricuspid regurgitation	Mechanical valve replacement	Not specified
Bai et al. (26)	2015	20/F	SLE	Acute right heart failure	Bioprosthetic valve replacement	Alive at 3 months
Waldoch et al. (27)	2016	13/F	SLE, APS	Cardiac mass (15×10mm)	Medical (steroids + anticoagulation)	Mass regression
Unic et al. (28)	2017	47/F	SLE, APS	Dyspnea, peripheral edema	Bioprosthetic valve replacement	Long-term asymptomatic
Mahajan et al. (29)	2017	34/F	APS	TIA, miscarriages, vegetation (6×8mm)	Bioprosthetic valve replacement	NYHA I at 12 months
Unnikrishnan et al. (30)	2018	60/F	APS	Fever, embolism	Mechanical valve replacement	NYHA II at 6 months
Yordan-Lopez et al. (31)	2018	53/M	APS	Heart failure, right heart mass	Bioprosthetic valve replacement	Improved
Nagi et al. (32)	2022	44/F	SLE, APS	Severe tricuspid regurgitation	Medical (anticoagulation + HCQ)	Symptom relief
Migliorini et al. (33)	2022	38/F	APS	Pregnancy (15 weeks), tricuspid regurgitation	Bioprosthetic valve replacement	C-section at 35 weeks
Lu et al. (34)	2024	14/F	SLE	Tricuspid regurgitation	Medical (steroids + MMF + rituximab, etc.)	Vegetation resolved at 28 days
Velasquez-Orozco et al. (35)	2025	41/F	SLE, APS	Fever, SAH, severe tricuspid regurgitation	Bioprosthetic valve replacement	NYHA I at 3 months
Present case	2026	21/F	SLE, APS	Pregnancy (13 weeks), severe thrombocytopenia, two vegetations (largest 24.1×19.3 mm)	Medical (immunosuppression + PLEX + anticoagulation)	Vegetation regression, healthy delivery

F, female; M, male; MMF, mycophenolate mofetil; SAH, subarachnoid hemorrhage; TIA, transient ischemic attack.

In contrast, our patient presented with a more complex and life-threatening profile: SLE with secondary APS, two tricuspid vegetations with the largest measuring 24.1 × 19.3 mm, severe thrombocytopenia with active bleeding, and moderate SLE disease activity that was compounded by life-threatening hematologic and cardiac manifestations during pregnancy. To address this critical situation, we opted for intensive immunomodulatory therapy, including early and aggressive use of PLEX, high-dose glucocorticoids, and IVIG. Most immunosuppressants and biologics carry teratogenic risks, substantially limiting treatment options during pregnancy; we therefore selected safer alternatives, including CsA and HCQ (36). Mechanistically, PLEX rapidly removes pathogenic autoantibodies, immune complexes, and inflammatory mediators, and is particularly useful in critically ill SLE patients (37). A study of 14 pregnant SLE patients by Zhang et al. demonstrated significantly better outcomes with PLEX: all 7 treated patients delivered successfully, compared with 3 miscarriages among 7 controls (37). In our patient, combination therapy with high-dose glucocorticoids and IVIG raised the platelet count from 22 × 10^9^/L to 63 × 10^9^/L, and five sessions of PLEX subsequently normalized it. PLEX was performed using fresh frozen plasma as the replacement fluid. During each session, maternal vital signs were closely monitored because hypotension—a known complication of plasma exchange—may reduce uteroplacental perfusion and precipitate fetal bradycardia. Isovolemic replacement was maintained to ensure hemodynamic stability, and continuous fetal heart rate auscultation was performed throughout each procedure. Serial electrolyte monitoring, with particular attention to ionized calcium levels, was conducted given the risk of citrate-induced hypocalcemia. No maternal hemodynamic instability, electrolyte disturbance, allergic reaction, or fetal distress occurred during any of the five sessions. A key management challenge was the timing of anticoagulation therapy. Severe thrombocytopenia (platelet count < 50 × 10^9^/L) is conventionally considered a relative contraindication to therapeutic anticoagulation due to bleeding risk. However, this view has been challenged in APS: studies have shown that APS patients with thrombocytopenia have a higher risk of recurrent thromboembolic events than those with normal platelet counts (2, 9). Although our patient’s initial platelet count was only 21 × 10^9^/L, the 24 mm vegetation posed an immediate threat of pulmonary embolism if dislodged. Once the platelet count rose to 36 × 10^9^/L and active bleeding resolved, the MDT judged that the thromboembolic risk outweighed the bleeding risk, and low-molecular-weight heparin was cautiously initiated at a therapeutic dose. When the PLT exceeded 50 × 10^9^/L, LDA was added. This strategy successfully avoided both major bleeding and thromboembolic complications, supporting the safety and necessity of early anticoagulation following partial platelet recovery in high-risk patients. This combined approach ultimately eliminated the need for cardiac surgery during pregnancy.

This case offers three key clinical lessons. First, in SLE/APS patients presenting with right-sided valvular vegetations and negative blood cultures, LSE should be the primary diagnostic consideration, and non-invasive imaging plays a key role in diagnosis (38). Second, intensive immunomodulatory therapy (including PLEX, high-dose glucocorticoids, IVIG, CsA, and HCQ) combined with timely and balanced anticoagulation can effectively control disease activity, promote vegetation regression, and avoid high-risk cardiac surgery. In our patient, the timing of anticoagulation was adapted based on real-time changes in bleeding status, platelet count, and serial echocardiographic findings, reflecting a flexible, risk-adapted approach rather than rigid adherence to conventional contraindications. Third, multidisciplinary collaboration among rheumatologists, cardiac surgeons, obstetricians, ultrasonographers, and hematologists is indispensable for the successful management of complex pregnancies in patients with SLE/APS. Several limitations should be acknowledged. First, follow-up was limited to the perinatal period; long-term monitoring of valvular function and vegetation recurrence is needed. Second, LA testing was not locally available, which precluded complete laboratory characterization of her aPLs profile. Third, pathological confirmation was not obtained, an inherent limitation of all non-surgically managed LSE cases.

## Conclusion

In conclusion, this case demonstrates that intensive immunomodulatory therapy combined with timely anticoagulation can achieve significant vegetation regression and avoid high-risk cardiac surgery in pregnant patients with SLE/APS and massive isolated tricuspid LSE. The strategy of initiating anticoagulation after partial platelet recovery provides a practical framework for balancing bleeding and thromboembolic risks in similar high-risk scenarios. This approach underscores the potential of non-surgical management for life-threatening cardiac manifestations of SLE/APS during pregnancy.

### Patient perspective

The patient provided the following perspective: “When I was told that I had a very low platelet count and a large mass on my heart valve during my pregnancy, I was terrified. The doctors explained that both the bleeding risk and the risk of the mass causing a lung clot were very high. They recommended ending the pregnancy and considering heart surgery, which I could not accept. I decided to continue the pregnancy and trust the medical team’s plan for aggressive medication. The treatment process was hard, but I felt my symptoms gradually improve. I am overjoyed that my baby was born healthy and that the mass on my valve has shrunk so much without needing surgery. I am deeply grateful to the entire team for their care and for respecting my decision.”

## Data Availability

The original contributions presented in the study are included in the article/**Supplementary Material**. Further inquiries can be directed to the corresponding authors.
